# Losartan, but not Enalapril and Valsartan, Inhibits the Expression of IFN-γ, IL-6, IL-17F and IL-22 in PBMCs from Rheumatoid Arthritis Patients

**DOI:** 10.2174/1874312901812010160

**Published:** 2018-09-18

**Authors:** Pablo R. G. Cardoso, Katherine A. Matias, Andrea T. Dantas, Claudia D. L. Marques, Michelly C. Pereira, Angela L. B. P. Duarte, Moacyr Jesus Barreto de Melo Rego, Ivan da Rocha Pitta, Maira Galdino da Rocha Pitta

**Affiliations:** 1 Laboratory of Immunomodulation and New Therapeutic Approaches (LINAT), Nucleus of Research in Immunomodulation and New Therapeutic Approaches Suely Galdino (Nupit SG), Federal University of Pernambuco (UFPE), Recife, Brazil; 2 Rheumatology Service, Hospital das Clínicas, Federal University of Pernambuco, Recife, Brazil

**Keywords:** Cytokines, Immunomodulatory effects, Therapy, Systemic Arterial Hypertension, Cardiovascular Disease, Rheumatology

## Abstract

**Background:**

Rheumatoid Arthritis (RA) is a chronic and inflammatory disease that affects about 1% of the world's population. Almost 70% of RA patients have a cardiovascular disease such as Systemic Arterial Hypertension (SAH). Inflammatory cytokines are clearly involved in the pathogenesis of RA and correlated with SAH.

**Objective:**

It is necessary to understand whether the antihypertensive drugs have a dual effect as immunomodulators and which one is the best choice for RA SAH patients.

**Methods:**

Peripheral Blood Mononuclear Cells (PBMCs) from 16 RA patients were purified and stimulated or not stimulated with anti-CD3 and anti-CD28 mAB and were treated with Enalapril, Losartan and Valsartan at 100μM. Patients were evaluated for clinical and laboratory variables including measures of disease activity by Clinical Disease Activity Index (CDAI) and Disease Activity Score (DAS28). Cytokines were quantified by ELISA sandwich.

**Results:**

Losartan was able to reduce levels of IFN-γ (*p* = 0.0181), IL-6 (*p* = 0.0056), IL-17F (0.0046) and IL-22 (*p* = 0.0234) in RA patients. In addition, patients in remission and mild score (DAS28<3.2 and CDAI<10) had a better response to treatment. On the other hand, patients in moderate and severe activity had poor response to Losartan in cytokine inhibition.

**Conclusion:**

PBMCs from RA patients are responsive in inhibiting proinflammatory cytokines using Losartan better than Enalapril and Valsartan and it could be a better antihypertensive choice for patients with RA and systemic arterial hypertension treatment.

## 1 INTRODUCTION

Rheumatoid Arthritis (RA) is a chronic and systemic disease that affects the joints, connective tissues, muscles, tendons and fibrous tissues, and in addition to pain, causing deformations in the bony-cartilaginous structure. The prevalence is approximately 1% of the world population [1]. The RA is mediated by Th1/Th17 cells, where TNF, IL-6 and IL-17 cytokines are key components in the RA pathogenesis [2].

Cardiovascular disease (CVD) represents one of the most frequent comorbidities in RA patients, and it is responsible for increasing the mortality of this population up to twice [3, 4]. A recent study indicates that in several countries, cardiovascular diseases remain the main comorbidities found in patients with RA (4). The prevalence of systemic arterial hypertension (SAH) is high in patients with RA, ranging from 53% -73% [5]. When comparing this percentage with the general population, this index is no more than 23% [5]. It is also known that hypertension is associated with the presence of asymptomatic lesions in RA patients regardless of the level of inflammatory activity [6]. With this, the need has become evident for a better understanding and attention at the time medication is prescribed for patients with RA [7].

Recent studies have highlighted the role of inflammation in the development of hypertension. Mechanisms of innate immunity, effector and regulatory T cells, and costimulatory molecules seem to be involved in the ethiopatogenesis of hypertension, interacting mostly with the Renin-Angiotensin-Aldosterone System (RAAS). This is the main responsible agent for the regulation of blood pressure and water balance [8]. It is suggested that the RAAS has a relationship with the activation of the immune system once there is more circulating aldosterone, activating angiotensin type 1 (AT1) receptors in immune cells such as lymphocytes and monocytes [9, 10].

There is a consensus of the Brazilian Society of Rheumatology regarding the treatment of comorbidities in RA. For SAH, the indicated treatment is composed of Angiotensin Converting Enzyme (ACE) inhibitors and/or angiotensin II receptor blockers (ARBs), preferably [11].

In a study with rats on an experimental autoimmune myocarditis and treated with Valsartan, there was a suppression of Th1 serum cytokine levels and significant positive regulation of Th2 cytokines, demonstrating the benefits of Valsartan as a balance modulator of Th subgroups [12]. Some studies point to Enalapril as a weak immunomodulator with few effects on the cytokine production by macrophages [13, 14]. On the other hand, Losartan presented better results being able to reduce IL-6 levels and increasing TGF-β production in patients with cardiovascular diseases [15, 16].

Valsartan and Telmisartan were tested in type II diabetes with immune effects, perhaps binding to Peroxisome Proliferator-Activated Receptor Gamma (PPAR-γ) [17], and Valsartan is able to slow down the progression of chronic kidney disease [18]. In adult patients with Marfan syndrome, Losartan as treatment reduced the rate of dilation of the aortic arch [19]. In addition, Losartan and Enalapril were capable of reducing the expression of IL-1β in macrophages [20] and polymorphonuclear leukocytes from patients with essential hypertension [21].

Clearly ARBs and ACEs have different effects besides blood pressure control, but their immunomodulatory effects in PBMCs from RA patients are still unclear. The objective of the present study was to evaluate the effects of Enalapril, Losartan and Valsartan on the production of IL-2, IL-10, IL-6, TNF, IFN-γ, IL-17A, IL-17F and IL-22 cytokines in PBMCs of patients with rheumatoid arthritis and evaluate this modulation with disease activity.

## 2 METHODS

### 2.1 Volunteers Recruitment

Sixteen RA patients were invited and recruited from Rheumatology Division at Clinics Hospital – Federal University of Pernambuco, Brazil, to participate. Demographic and clinical parameters were collected from all patients by questionnaire (Table **1**). DAS28 and CDAI were measured and criteria for RA classification [22, 23]. A DAS28 lower than 3.2 implied low disease activity, between 3.2–5.1 implied moderate and higher than 5.1 meant high disease activity, while CDAI less than 2.8 implied clinical remission, lower than 10 implied mild activity, between 10–22 implied moderate and finally higher than 22 score implied severe activity.

The presence of other rheumatic diseases, cardiovascular, endocrine and thyroid, immunomodulated and cancer diseases were exclusion criteria for all volunteers. Moreover, being under biological, immunomodulatory or antihypertensive treatments also were exclusion criteria. The study was approved by the Federal University of Pernambuco (UFPE) ethics committee (CAAE: 53555116.0.0000.5208).

### 2.2 Antihypertensives Drugs

We used 3 antihypertensive drugs. Two of them are ARBs, Losartan and Valsartan and an ACE Enalapril. All medicines were chosen because they are widely used in cardiovascular diseases around the world. For a better understanding, the drugs were named using the initials: Enalapril (E), Losartan (L) and Valsartan (V).

### 2.3 PBMCs Sample for Immunomodulatory Experiments

Peripheral Blood Mononuclear Cells (PBMC) from RA volunteers were obtained using a heparinized tube and isolated by density-gradient centrifugation with Ficoll-Hypaque (GE Healthcare). Therefore, $1\times 10^{6}$ cells/ml were disposed in RPMI-1640 media (Gibco) supplemented with 10% fetal bovine serum (Gibco), HEPES 10 mM (Gibco) and penicillin/streptomycin 200 U/ml (Gibco).

Cells were stimulated using anti-CD3 and anti-CD28 Monoclonal Antibodies (mAB) (eBioscience) - once RA is a T-lymphocyte-mediated disease – [24, 25] in the presence or absence of Losartan, Valsartan and Enalapril at 100μM. Methylprednisolone at 100μM was used as a standard drug. Cells were incubated for 48hours at 37°C in humidified 5% CO2 incubator, therefore the supernatant was collected and stocked at -80°C for posterior dosages.

### 2.4 Cytokine Measurement

Enzyme-linked Immunosorbent Assay (ELISA) kits according to the manufacturer’s instructions determined Cytokines present in the supernatants. The detection limits for TNF, IL-10, IL-6 and IFN-γ (BD Biosciences), IL-2, IL-17A, IL-17F and IL-22 (eBioscience) were 3.90, 3.90, 4.68, 4.68, 1.95, 3.90, 15.62 and 7.81 pg/ml, respectively.

### 2.5 Statistical Analysis

The Wilcoxon and Student’s t tests were used for statistical analysis and *p* values < 0.05 were statistically significant. Values are expressed as the median, maximum and minimum for supernatant dosages and mean ± Standard Deviation (SD) for demographic parameters. All quantitative data was plotted with GraphPad Prism^®^ 6.01.

## 3 RESULTS

### 3.1 RA Patients Parameters

A total of 16 patients fulfilled four or more American College of Rheumatology (ACR) 2010 diagnostic criteria [26]. Individual disease activity was quantified using the DAS28 [23] and CDAI [22]. Demographic, clinical and laboratory data were collected, and these results are shown in Table **1**.

### 3.2 Enalapril, Losartan and Valsartan Inhibiting Inflammatory Cytokines

The first step was to evaluate the cytotoxicity of the antihypertensives Enalapril (E), Losartan (L) and Valsartan (V) in PBMC to confirm a non-toxic concentration. We tested five different concentrations (10, 25, 50, 75 and 100µM) in triplicate of concentration and triplicate of experiment. None of the drugs tested showed toxicity at the used concentration and viability was higher than 98% (data not shown) and the 100μM was used for tests.

Cytokines levels can be seen in Fig. (**1**). Median, maximum and minimum expresses the results. There was a significant decrease in IFN-γ by Enalapril at 100µM [2988.53 (18895.29-4.68)] (*p*= 0.0302) compared to the stimulated condition [5157.895 (63637.65-155.29)] and IL-22 [13.65 (195.58-7.81)] compared to mAB stimulation only [100.84 (218.67-9.50)] (*p*=0.0161).

Losartan had immunomodulatory effects reducing IFN-γ, IL-6, IL-17F and IL-22. For IFN-γ, there was significant decrease by Losartan at 100μM [2252.6 (20461.1-4.6)] compared to the stimulated condition (p = 0.0181). For IL-6 it was [2154.2 (8480.8-4.6)] compared to the stimulated condition [3178.60 (10064.8-136.4)] (*p* = 0.0056). We also found a significant reduction in IL-17F by Losartan [457.6 (1239.8-129.6)] compared to the stimulated condition [1181.5 (3836.5-236.0)] (*p* = 0.0046). Finally, IL-22 had a significant reduction by Losartan in RA group [100.4 (175.5-7.8)] compared to the stimulated condition [100.84 (218.67-9.50)] (*p* = 0.0234). Valsartan showed no significant association.

### 3.3 Effect of Antihypertensive Drugs on Cytokine Profile Association with Disease Activity of RA

**Fig. (2):**
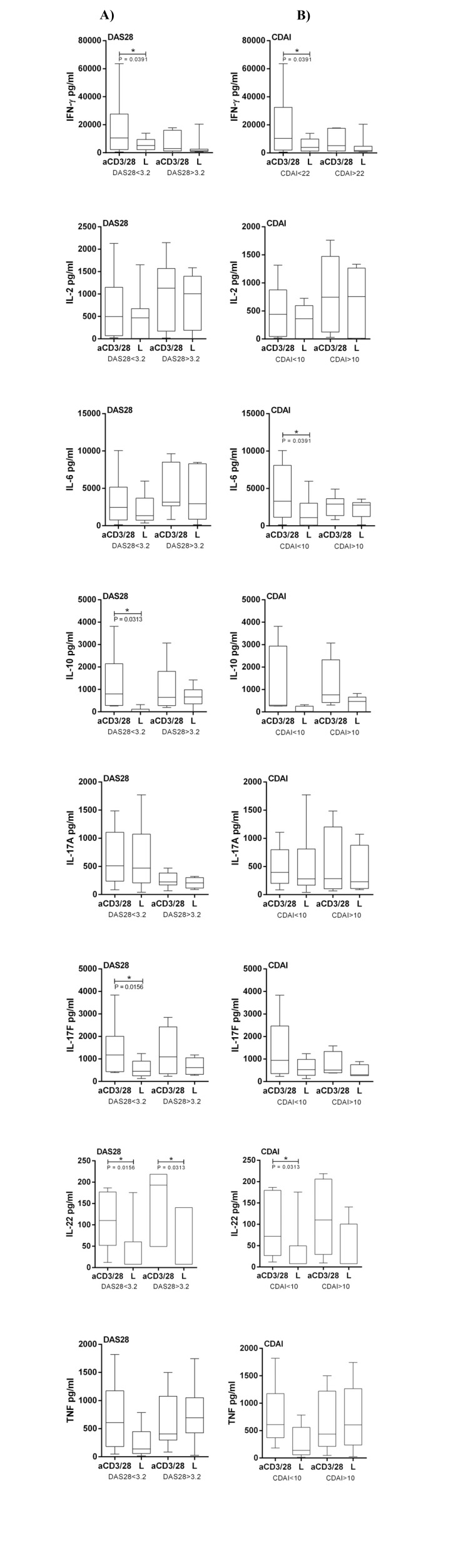
Cytokine levels from patients with rheumatoid arthritis treated with 100μM of Losartan and a Disease Activity Score for 28 joints (a IFN-γ; IL-2; IL-6; IL-17A; IL-17F; IL-22; TNF) and a Clinical Disease Activity Index (b IFN-γ; IL-2; IL-6; IL-17A; IL-17F; IL-22; TNF).

Subsequently, we analyzed the effect of Enalapril, Losartan and Valsartan on the cytokine profile and evaluated if there was any correlation with disease activity measured by DAS28 and CDAI. There was significant association for IFN-γ, IL-10, IL-17F and IL-22 according DAS28 < 3.2 (*p* = 0.0391; 0.0313; 0.0156; 0.0156 respectively) and for DAS28 > 3.2 in IL-22 reduction (*p* = 0,0313) after 100μM of Losartan treatment (Fig. **2A**). In concurrence, there was significant association for IFN-γ, IL-6 and IL-22 also according CDAI < 10 (*p* = 0.0391; 0.0391; 0.0313 respectively) after 100μM of Losartan treatment (Fig. **2B**). No association was found in Enalapril and Valsartan treatments.

It was observed that RA patients with severe disease had a worse response to the Losartan in reducing cytokines levels. The same profile was observed for other cytokines, however without significance.

## 4 DISCUSSION

Experimental *in vitro* and *in vivo* studies have strongly demonstrated that antihypertensive drugs have anti-inflammatory and immunomodulatory functions [8, 12, 15, 27]. However, the potential of these drugs in rheumatoid arthritis disease has not yet been demonstrated. We decided to analyze the effect of anti-hypertensive drugs in PBMCs variations corresponding to disease activity by DAS28 and CDAI according to the international criteria.

RA develops and progresses with the immune system imbalance involving defense cells, mainly by B cells and CD4+ T cells and their subgroups. The most involved T helper in RA pathogenesis are Th1 and Th17 and their inflammatory cytokines produced [28]. RA patients generally suffer with cardiovascular diseases - incidence is higher than 70% (5). Immunological markers of inflammation are expressed in higher concentrations in RA patients who have CVC diseases, including highly sensitive C-reactive protein [29].

For SAH in RA, the indicated treatment is composed of ACE and/or ARBs, preferably (11). ACE inhibitors and ARBs are used in the treatment of cardiovascular diseases with good results, including for RA patients [30]. Cardiovascular risk factors and systemic inflammation are associated with the rapid progression of carotid intima-media thickness in RA patients [31]. For this reason, it is necessary to test whether the antihypertensive drugs promote immunomodulation and if one of them can be a first decision for the SAH treatment in RA patients.

Our results show that Losartan at 100µM could significantly reduce IFN-γ, IL-6, IL-17F, IL-22 cytokine levels secreted by PBMCs in RA cultures. The other antihypertensive drugs tested, Enalapril and Valsartan had few or no immunomodulatory effects on the production of proinflammatory cytokines in PBMCs.

We showed that Losartan consistently reduced IL-17F cytokine levels in a 100µM concentration. In addition, IL-17F correlates with disease severity by DAS28 and CDAI, Losartan was able to sensitize patients' cells in remission/mild/moderate RA condition reducing the levels of this cytokine, but no effects were seen in severe score.

Proinflammatory cytokines are involved in RA and aggravate the disease development. One of them is IL-17. IL-17A and IL-17F are highly reported in RA and contribute to the RA pathogenesis [32]. Both IL-17A and IL-17F are present in synovium and aggravate the illness [28]. Accordingly, IL-17F appears as a target in Th17-mediated diseases such as RA [33, 34]. That is interesting because although involved in RA, IL-17 also plays a key role in the pathogenesis of arterial hypertension, inducing the production of angiogenic factors and migration of endothelial cells [35]. IL-17A also mediates Angiotensin II (Ang II) induced renal injury and regulates renal sodium transporters, activating a serum and glucocorticoid regulated kinase 1 dependent pathway, and it could cause a sodium and water imbalance and therefore elevating the blood pressure [35-37]. Reducing IL-17 circulating levels would not only have an improvement in RA but would also benefit hypertensive patients. As we demonstrated, our results show a potential immunoregulation of Losartan in reducing IFN-γ levels. In addition to this Losartan was also able to statistically reduce significant IL-6 levels in PBMC culture from RA patients.

IFN-γ and IL-6 are two inflammatory cytokines with innumerable functions, such as cell proliferation and immune system regulation. IL-6 also is a treatment target for RA [38]. Cell responses from both are activated* via *Janus Kinase/Signal Transducer and Activator of Transcription Proteins (JAK/STAT) pathway [39]. The IFN family is related to the pathogenesis of RA and this is well described [40, 41]. This cytokine undoubtedly participates in the joint inflammation promoting cell recruitment and proliferation. IFN-γ is also considered a marker for RA [42]. IFN-γ and IL-6, although distinct, act together and can be produced in parallel in an inflammation site* via *similar transcription factors such as Interferon Regulatory Factor 1 (IRF-1) and intercellular adhesion molecule 1 (ICAM-1) [43]. In addition, IL-6 is also crucial in the pathogenesis of RA [44]. It is correlated with swollen joint count, c-reactive protein levels and associated with DAS28 severity, rheumatoid factor and serology of anti-citrullinated peptide antibodies [45].

Our results suggest that Losartan could significantly reduce the IL-22 levels produced by PBMC from RA patients. IL-22 is a pleiotropic cytokine. It is related to osteoclastogenesis by positively regulating the expression of Receptor Activator of Nuclear factor kappa-Β ligand (RANKL) mediated* via *Mitogen Activated Protein Kinase (MAPK) and Factor Nuclear kappa B - p38 MAPK/NF-κB and JAK-2/STAT-3 - signaling pathways [46]. But at the same time CD4 + Th22 cells are also related to the pathogenesis of RA, contributing to the worsening of this disease [47] by promoting the proliferation of synoviocytes [48], and may become a therapeutic target soon [49-51]. Few studies have correlated the levels of IL-22 with the RA severity and possible treatments to reduce this cytokine production [40, 52, 53].

For all cytokines evaluated we observed that patients with severe activity of disease (DAS28 > 3.2 and CDAI > 10) respond in worse efficiency to Losartan treatment. Patients in severe conditions probably have their cells sensitized due to an anti-inflammatory treatment and this makes the *in vitro* study harder. As RA is a chronic and degenerative disease, nowadays it is very difficult to recruit patients in severe conditions who are not taking any anti-inflammatory medication. A previously analysis was not performed to affirm if patients who were on Prednisone treatment could be an influencing factor.

Of the three antihypertensive drugs tested, Losartan improved immunomodulatory effects in PBMCs of patients with RA. Probably, this reduction is due to an inhibitory effect of Losartan on NF-κB upregulated in RA patients modulating proinflammatory cytokines [54, 55].

## CONCLUSION

In conclusion, our findings suggest that the Losartan therapy does modulate IFN-γ, IL-6, IL-17F and IL-22 at 100µM in PBMCs from RA patients according to disease activity. Moreover, none significative immunomodulatory activities were observed with Enalapril or Valsartan at 100µM in cytokine production, suggesting Losartan could be a better option for SAH treatment in RA patients. Further studies are still needed to elucidate the molecular mechanism that Losartan acts in PBMCs.

## Figures and Tables

**Fig. (1):**
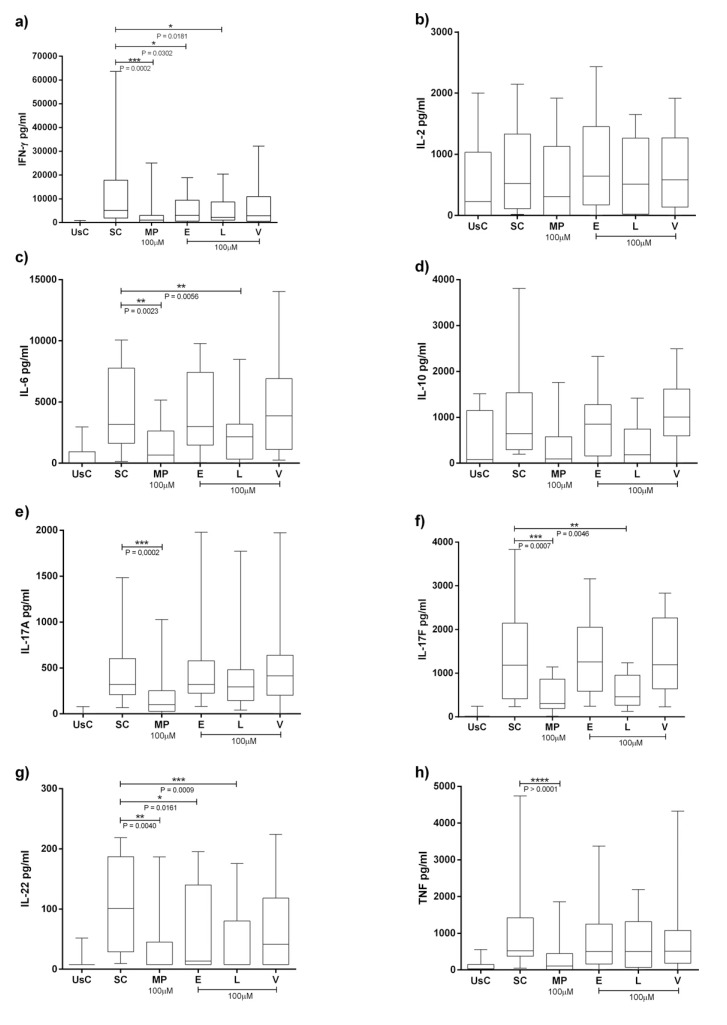
*In vitro* effects of Enalapril, Losartan and Valsartan after stimulation on PBMCs and their cytokines in RA. PBMCs from RA patients following stimulation with anti-CD3 and anti-CD28 mAB (SC) were treated with different conditions, Enalapril (E), Losartan (L) and Valsartan (V) at 100µM and Methylprednisolone (MP) at 100µM as standard drug. Cytokine concentrations of Th1/Th2/Th17 type cytokines were measured by ELISA in supernatants of PBMCs cultures from RA patients. A significant reduction in the IFN-γ and IL-22 concentration was detected in cultures of RA patients with Enalapril. Losartan reduced the concentration of IFN-γ; IL-6; IL-17F and IL-22 in PBMCs from RA patients. IL-2, IL-10 and IL-17A culture supernatants revealed no significant changes. Valsartan did not reduce any concentration in cytokines. (* = *p* <0.05; ** = *p* < 0.01; *** = *p* < 0.001 and **** = *p*< 0.0001).

**Table 1:** Clinical parameters of RA patients recruited.

**Characteristics** **Patients (n = 16)**	**Data**
**Female n**	14
**Male n**	2
**Age (years), mean (min-max)**	53 (32 – 73)
**Disease Duration (years), mean (range)**	10 (±7,9)
**DAS28 (%)** **Low Disease Activity (≤3.2)** **Moderate and High Disease Activity (>3.2)**	9 (56%) 7 (44%)
**CDAI (%)** **Low Disease Activity (≤ 10)** **Moderate and High Disease Activity (> 10)**	8 (50%) 8 (50%)
Treatment (%)* None **Prednisone** **Methotrexate** **Leflunomide**	3 9 11 2
**ESR mean (range mm/h)**	16 (±10)
**Rheumatoid Factor (%)**	16 (100%)
* Eight patients were on Prednisone and Methotrexate combination and only one on Leflunomide and Methotrexate combination.	

## LIST OF ABBREVIATIONS

(RA): = Rheumatoid Arthritis
(CVD): = Cardiovascular disease
(SAH): = Systemic Arterial Hypertension
(RAAS): = Renin-Angiotensin-Aldosterone System
(AT1): = Activating Angiotensin type 1
(E): = Enalapril
(L): = Losartan
(V): = Valsartan
(PBMCs): = Peripheral Blood Mononuclear Cells
(mAB): = Monoclonal Antibodies
(ELISA): = Enzyme-Linked Immunosorbent Assay
(SD): = Standard Deviation
(ACR): = American College of Rheumatology
(DAS28): = Disease Activity Score
(CDAI): = Clinical Disease Activity Index
(SC): = Stimulated Cells
(UsC): = Unstimulated Cells
(Th): = T helper

## References

[1] Vyas S., Bhalla A.S., Ranjan P., Kumar S., Kumar U., Gupta A.K. (2016). Rheumatoid arthritis revisited - Advanced imaging review.. Pol. J. Radiol..

[2] Hwang S.Y., Kim J.Y., Kim K.W., Park M.K., Moon Y., Kim W.U., Kim H.Y. (2004). IL-17 induces production of IL-6 and IL-8 in rheumatoid arthritis synovial fibroblasts* via *NF-kappaB- and PI3-kinase/Akt-dependent pathways.. Arthritis Res. Ther..

[3] Crepaldi G., Scirè C.A., Carrara G., Sakellariou G., Caporali R., Hmamouchi I., Dougados M., Montecucco C. (2016). Cardiovascular comorbidities relate more than others with disease activity in rheumatoid arthritis.. PLoS One.

[4] Dougados M. (2016). Comorbidities in rheumatoid arthritis.. Curr. Opin. Rheumatol..

[5] Kitas G.D., Gabriel S.E. (2011). Cardiovascular disease in rheumatoid arthritis: State of the art and future perspectives.. Ann. Rheum. Dis..

[6] Midtbø H., Gerdts E., Kvien T.K., Olsen I.C., Lønnebakken M.T., Davidsen E.S., Rollefstad S., Semb A.G. (2016). The association of hypertension with asymptomatic cardiovascular organ damage in rheumatoid arthritis.. Blood Press..

[7] van Breukelen-van der Stoep D.F., van Zeben D., Klop B., van de Geijn G.J., Janssen H.J., van der Meulen N., De Vries M.A., Hazes M., Birnie E., Castro Cabezas M. (2016). Marked underdiagnosis and undertreatment of hypertension and hypercholesterolaemia in rheumatoid arthritis.. Rheumatology (Oxford).

[8] Maeda A., Okazaki T., Inoue M., Kitazono T., Yamasaki M., Lemonnier F.A., Ozaki S. (2009). Immunosuppressive effect of angiotensin receptor blocker on stimulation of mice CTLs by angiotensin II.. Int. Immunopharmacol..

[9] Weber J., Tiriveedhi V., Takenaka M., Lu W., Hachem R., Trulock E., Patterson G.A., Mohanakumar T. (2012). Inhibition of renin angiotensin aldosterone system causes abrogation of obliterative airways disease through inhibition of tumor necrosis factor-α-dependant interleukin-17.. J. Heart Lung Transplant..

[10] Sanchez-Lemus E., Murakami Y., Larrayoz-Roldan I.M., Moughamian A.J., Pavel J., Nishioku T., Saavedra J.M. (2008). Angiotensin II AT1 receptor blockade decreases lipopolysaccharide-induced inflammation in the rat adrenal gland.. Endocrinology.

[11] Pereira I.A., Mota L.M., Cruz B.A., Brenol C.V., Fronza L.S., Bertolo M.B., Freitas M.V., Silva N.A., Louzada-Junior P., Giorgi R.D., Lima R.A., Pinheiro Gda.R. (2012). 2012 Brazilian society of rheumatology consensus on the management of comorbidities in patients with rheumatoid arthritis.. Rev. Bras. Reumatol..

[12] Liu X., Zhu X., Wang A., Fan H., Yuan H. (2009). Effects of angiotensin-II receptor blockers on experimental autoimmune myocarditis.. Int. J. Cardiol..

[13] Krysiak R., Okopień B. (2012). Different effects of perindopril and enalapril on monocyte cytokine release in coronary artery disease patients with normal blood pressure.. Pharmacol. Rep..

[14] Albuquerque D., Nihei J., Cardillo F., Singh R. (2010). The ACE inhibitors enalapril and captopril modulate cytokine responses in Balb/c and C57Bl/6 normal mice and increase CD4(+)CD103(+)CD25(negative) splenic T cell numbers.. Cell. Immunol..

[15] Sepehri Z., Masoumi M., Ebrahimi N., Kiani Z., Nasiri A.A., Kohan F., Sheikh Fathollahi M., Kazemi Arababadi M., Asadikaram G. (2016). Atorvastatin, losartan and captopril lead to upregulation of TGF-β, and downregulation of IL-6 in coronary artery disease and hypertension.. PLoS One.

[16] Sadamatsu K., Shimokawa H., Tashiro H., Seto T., Kakizoe H., Yamamoto K. (2006). Different effects of simvastatin and losartan on cytokine levels in coronary artery disease.. Am. J. Cardiovasc. Drugs.

[17] Miura Y., Yamamoto N., Tsunekawa S., Taguchi S., Eguchi Y., Ozaki N., Oiso Y. (2005). Replacement of valsartan and candesartan by telmisartan in hypertensive patients with type 2 diabetes: Metabolic and antiatherogenic consequences.. Diabetes Care.

[18] Yasuda T., Endoh M., Suzuki D., Yoshimura A., Ideura T., Tamura K., Kamata K., Toya Y., Umemura S., Kimura K., KVT Study Group (2013). Effects of valsartan on progression of kidney disease in Japanese hypertensive patients with advanced, predialysis, chronic kidney disease: Kanagawa Valsartan Trial (KVT).. Hypertens. Res..

[19] Groenink M., den Hartog A.W., Franken R., Radonic T., de Waard V., Timmermans J., Scholte A.J., van den Berg M.P., Spijkerboer A.M., Marquering H.A., Zwinderman A.H., Mulder B.J. (2013). Losartan reduces aortic dilatation rate in adults with Marfan syndrome: A randomized controlled trial.. Eur. Heart J..

[20] Hernández-Fonseca J.P., Durán A., Valero N., Mosquera J. (2015). Losartan and enalapril decrease viral absorption and interleukin 1 beta production by macrophages in an experimental dengue virus infection.. Arch. Virol..

[21] Nemati F., Rahbar-Roshandel N., Hosseini F., Mahmoudian M., Shafiei M. (2011). Anti-inflammatory effects of anti-hypertensive agents: Influence on interleukin-1β secretion by peripheral blood polymorphonuclear leukocytes from patients with essential hypertension.. Clin. Exp. Hypertens..

[22] Aletaha D., Nell V.P., Stamm T., Uffmann M., Pflugbeil S., Machold K., Smolen J.S. (2005). Acute phase reactants add little to composite disease activity indices for rheumatoid arthritis: validation of a clinical activity score.. Arthritis Res. Ther..

[23] Prevoo M.L., van ’t Hof M.A., Kuper H.H., van Leeuwen M.A., van de Putte L.B., van Riel P.L. (1995). Modified disease activity scores that include twenty-eight-joint counts. Development and validation in a prospective longitudinal study of patients with rheumatoid arthritis.. Arthritis Rheum..

[24] Trickett A., Kwan Y.L. (2003). T cell stimulation and expansion using anti-CD3/CD28 beads.. J. Immunol. Methods.

[25] Colin E.M., Asmawidjaja P.S., van Hamburg J.P., Mus A.M., van Driel M., Hazes J.M., van Leeuwen J.P., Lubberts E. (2010). 1,25-dihydroxyvitamin D3 modulates Th17 polarization and interleukin-22 expression by memory T cells from patients with early rheumatoid arthritis.. Arthritis Rheum..

[26] Aletaha D., Neogi T., Silman A.J., Funovits J., Felson D.T., Bingham C.O., Birnbaum N.S., Burmester G.R., Bykerk V.P., Cohen M.D., Combe B., Costenbader K.H., Dougados M., Emery P., Ferraccioli G., Hazes J.M., Hobbs K., Huizinga T.W., Kavanaugh A., Kay J., Kvien T.K., Laing T., Mease P., Ménard H.A., Moreland L.W., Naden R.L., Pincus T., Smolen J.S., Stanislawska-Biernat E., Symmons D., Tak P.P., Upchurch K.S., Vencovsky J., Wolfe F., Hawker G. (2010). 2010 rheumatoid arthritis classification criteria: An American college of rheumatology/european league against rheumatism collaborative initiative.. Ann. Rheum. Dis..

[27] Wang F., Huang L., Peng Z.Z., Tang Y.T., Lu M.M., Peng Y., Mel W.J., Wu L., Mo Z.H., Meng J., Tao L.J. (2014). Losartan inhibits LPS + ATP-induced IL-1beta secretion from mouse primary macrophages by suppressing NALP3 inflammasome.. Pharmazie.

[28] Furst D.E., Emery P. (2014). Rheumatoid arthritis pathophysiology: Update on emerging cytokine and cytokine-associated cell targets.. Rheumatology (Oxford).

[29] Popkova T.V., Khelkovskaia A.N., Mach E.S., Aleksandrova E.N., Novikov A.A., Novikova D.S., Nasonov E.L. (2007). Cardiovascular diseases in rheumatoid arthritis.. Ter. Arkh..

[30] de Jong H.J., Vandebriel R.J., Saldi S.R., van Dijk L., van Loveren H., Cohen Tervaert J.W., Klungel O.H. (2012). Angiotensin-converting enzyme inhibitors or angiotensin II receptor blockers and the risk of developing rheumatoid arthritis in antihypertensive drug users.. Pharmacoepidemiol. Drug Saf..

[31] del Rincón I., Polak J.F., O’Leary D.H., Battafarano D.F., Erikson J.M., Restrepo J.F., Molina E., Escalante A. (2015). Systemic inflammation and cardiovascular risk factors predict rapid progression of atherosclerosis in rheumatoid arthritis.. Ann. Rheum. Dis..

[32] Zrioual S., Ecochard R., Tournadre A., Lenief V., Cazalis M.A., Miossec P. (2009). Genome-wide comparison between IL-17A- and IL-17F-induced effects in human rheumatoid arthritis synoviocytes.. J. Immunol..

[33] Paradowska-Gorycka A., Wojtecka-Lukasik E., Trefler J., Wojciechowska B., Lacki J.K., Maslinski S. (2010). Association between IL-17F gene polymorphisms and susceptibility to and severity of Rheumatoid Arthritis (RA).. Scand. J. Immunol..

[34] Hot A., Miossec P. (2011). Effects of interleukin (IL)-17A and IL-17F in human rheumatoid arthritis synoviocytes.. Ann. Rheum. Dis..

[35] Bean C., Spencer S.K., Bowles T., Kyle P.B., Williams J.M., Gibbens J., Wallace K. (2016). Inhibition of T-cell activation attenuates hypertension, TNFα, IL-17, and blood-brain barrier permeability in pregnant rats with angiogenic imbalance.. Am. J. Reprod. Immunol..

[36] Marder W., Khalatbari S., Myles J.D., Hench R., Yalavarthi S., Lustig S., Brook R., Kaplan M.J. (2011). Interleukin 17 as a novel predictor of vascular function in rheumatoid arthritis.. Ann. Rheum. Dis..

[37] Norlander A.E., Saleh M.A., Kamat N.V., Ko B., Gnecco J., Zhu L., Dale B.L., Iwakura Y., Hoover R.S., McDonough A.A., Madhur M.S. (2016). Interleukin-17A regulates renal sodium transporters and renal injury in angiotensin II-induced hypertension.. Hypertension.

[38] Ash Z., Emery P. (2012). The role of tocilizumab in the management of rheumatoid arthritis.. Expert Opin. Biol. Ther..

[39] Qi Y.F., Huang Y.X., Wang H.Y., Zhang Y., Bao Y.L., Sun L.G., Wu Y., Yu C.L., Song Z.B., Zheng L.H., Sun Y., Wang G.N., Li Y.X. (2013). Elucidating the crosstalk mechanism between IFN-gamma and IL-6* via *mathematical modelling.. BMC Bioinformatics.

[40] da Rocha Junior L.F., Rêgo M.J., Cavalcanti M.B., Pereira M.C., Pitta M.G., de Oliveira P.S., Gonçalves S.M., Duarte A.L., de Lima Mdo.C., Pitta Ida.R., Pitta M.G. (2013). Synthesis of a novel thiazolidinedione and evaluation of its modulatory effect on IFN- γ, IL-6, IL-17A, and IL-22 production in PBMCs from rheumatoid arthritis patients.. BioMed Res. Int..

[41] Scarsi M., Zanotti C., Chiarini M., Imberti L., Piantoni S., Frassi M., Tincani A., Airò P. (2014). Reduction of peripheral blood T cells producing IFN-γ and IL-17 after therapy with abatacept for rheumatoid arthritis.. Clin. Exp. Rheumatol..

[42] Lübbers J., Brink M., van de Stadt L.A., Vosslamber S., Wesseling J.G., van Schaardenburg D., Rantapää-Dahlqvist S., Verweij C.L. (2013). The type I IFN signature as a biomarker of preclinical rheumatoid arthritis.. Ann. Rheum. Dis..

[43] Yuan J., Wegenka U.M., Lütticken C., Buschmann J., Decker T., Schindler C., Heinrich P.C., Horn F. (1994). The signalling pathways of interleukin-6 and gamma interferon converge by the activation of different transcription factors which bind to common responsive DNA elements.. Mol. Cell. Biol..

[44] Calabrese L.H., Rose-John S. (2014). IL-6 biology: Implications for clinical targeting in rheumatic disease.. Nat. Rev. Rheumatol..

[45] Baillet A., Gossec L., Paternotte S., Etcheto A., Combe B., Meyer O., Mariette X., Gottenberg J.E., Dougados M. (2015). Evaluation of serum interleukin-6 level as a surrogate marker of synovial inflammation and as a factor of structural progression in early rheumatoid arthritis: Results from a French national multicenter cohort.. Arthritis Care Res. (Hoboken).

[46] Kim K.W., Kim H.R., Park J.Y., Park J.S., Oh H.J., Woo Y.J., Park M.K., Cho M.L., Lee S.H. (2012). Interleukin-22 promotes osteoclastogenesis in rheumatoid arthritis through induction of RANKL in human synovial fibroblasts.. Arthritis Rheum..

[47] Zhao L., Jiang Z., Jiang Y., Ma N., Zhang Y., Feng L., Wang K. (2013). IL-22+ CD4+ T cells in patients with rheumatoid arthritis.. Int. J. Rheum. Dis..

[48] Zhu J., Jia E., Zhou Y., Xu J., Feng Z., Wang H., Chen X., Li J. (2015). Interleukin-22 secreted by NKp44+ natural killer cells promotes proliferation of fibroblast-like synoviocytes in rheumatoid arthritis.. Medicine.

[49] Koenders M.I., van den Berg W.B. (2015). Novel therapeutic targets in rheumatoid arthritis.. Trends Pharmacol. Sci..

[50] Xie Q., Huang C., Li J. (2015). Interleukin-22 and rheumatoid arthritis: Emerging role in pathogenesis and therapy.. Autoimmunity.

[51] Xie Q., Wang S.C., Li J. (2012). Interleukin 22, a potential therapeutic target for rheumatoid arthritis.. J. Rheumatol..

[52] Pereira M.C., Cardoso P.R., Da Rocha L.F., Rêgo M.J., Gonçalves S.M., Santos F.A., Galdino-Pitta M.R., Dantas A.T., Duarte Â.L., Pitta M.G. (2014). Simvastatin inhibits cytokines in a dose response in patients with rheumatoid arthritis.. Inflamm. Res..

[53] da Rocha L.F., Duarte Â.L., Dantas A.T., Mariz H.A., Pitta Ida.R., Galdino S.L., Pitta M.G. (2012). Increased serum interleukin 22 in patients with rheumatoid arthritis and correlation with disease activity.. J. Rheumatol..

[54] Guo G., Cheng X., Fu R. (2013). Losartan inhibits nuclear factor-κB activation induced by small, dense LDL cholesterol particles in human umbilical vein endothelial cells.. Curr. Ther. Res. Clin. Exp..

[55] Pattacini L., Casali B., Boiardi L., Pipitone N., Albertazzi L., Salvarani C. (2007). Angiotensin II protects fibroblast-like synoviocytes from apoptosis* via *the AT1-NF-kappaB pathway.. Rheumatology (Oxford).

